# Randomly Multiplexed Diffractive Lens and Axicon for Spatial and Spectral Imaging

**DOI:** 10.3390/mi11040437

**Published:** 2020-04-21

**Authors:** Vijayakumar Anand, Tomas Katkus, Saulius Juodkazis

**Affiliations:** 1 Optical Sciences Centre and ARC Training Centre in Surface Engineering for Advanced Materials (SEAM), School of Science, Swinburne University of Technology, Hawthorn, VIC 3122, Australia; tkatkus@swin.edu.au; 2 Melbourne Centre for Nanofabrication, Australian National Fabrication Facility, 151 Wellington Road, Clayton, VIC 3168, Australia; 3 Tokyo Tech World Research Hub Initiative (WRHI), School of Materials and Chemical Technology, Tokyo Institute of Technology, 2-12-1, Ookayama, Meguro-ku, Tokyo 152-8550, Japan

**Keywords:** Fresnel zone lens, axicon, incoherent imaging, diffractive optics, three-dimensional imaging, spectral imaging, correlation optics

## Abstract

A new hybrid diffractive optical element (HDOE) was designed by randomly multiplexing an axicon and a Fresnel zone lens. The HDOE generates two mutually coherent waves, namely a conical wave and a spherical wave, for every on-axis point object in the object space. The resulting self-interference intensity distribution is recorded as the point spread function. A library of point spread functions are recorded in terms of the different locations and wavelengths of the on-axis point objects in the object space. A complicated object illuminated by a spatially incoherent multi-wavelength source generated an intensity pattern that was the sum of the shifted and scaled point spread intensity distributions corresponding to every spatially incoherent point and wavelength in the complicated object. The four-dimensional image of the object was reconstructed using computer processing of the object intensity distribution and the point spread function library.

## 1. Introduction

Fresnel incoherent correlation holography (FINCH) was invented in 2007 for the three-dimensional imaging of objects [1]. In FINCH, an object is illuminated using a spatially incoherent light source and the object wave is split into two using randomly multiplexed Fresnel zone lenses displayed on a spatial light modulator [1]. The two resulting mutually coherent object waves are made to interfere with each other to produce a self-interference hologram. Three such self-interference holograms were recorded corresponding to three different relative phase differences (θ = 0, 2π/3, 4π/3) between the two object waves and combined to produce a complex hologram to remove the twin image and bias terms during reconstruction using Fresnel backpropagation. In subsequent studies, the optical configuration of FINCH was upgraded to yield an improved performance [2,3,4,5]. Noise suppression techniques using statistical averaging [2] and a polarization multiplexing scheme [3] were introduced to remove the multiplexing noise in the reconstruction that was prevalent in the earlier random phase multiplexing method [1]. This procedure improved the signal-to-noise ratio of the reconstruction in FINCH. 

Later, the super-resolution capabilities of FINCH were uncovered and the optical configuration and conditions for achieving super-resolution were determined [4,5]. FINCH was able to break the Lagrange invariant condition and reach a lateral resolving power of 1.5 times as an equivalent incoherent imaging system with the same numerical aperture [4,5]. For this reason, FINCH became an attractive imaging tool for fluorescence microscopy [6] and for enhancing the resolving power of other existing super-resolution techniques, such as structured illumination [7], coded aperture imaging [8], and confocal imaging [9] techniques.

While the advantages of FINCH are evident, there are some drawbacks associated with the implementation of FINCH, which decreases the applicability spectrum of FINCH. The main drawback with FINCH is the need for at least three camera shots, which in turn requires a spatial light modulator (SLM) or an active device. Many researchers have tried to overcome this disadvantage in FINCH using ingenious techniques [10,11,12,13,14,15]. In Tahara et al. [10], a micropolarizer array with four orthogonal polarizations was used to obtain four phase shifts in the same intensity distribution. The intensity distribution was processed and interpolated to obtain four phase-shifted intensity patterns, which were projected into complex space and the complex hologram was synthesized. However, the reconstruction results from this method were noisy due to extensive computer processing. A similar approach, but instead of polarization multiplexing, a phase multiplexing method was introduced to encode four phase-shifted Fresnel zone lenses in the same phase mask, which were then displayed on the SLM [11]. The problem in this approach was that the field of view of the imaging was decreased as the image sensor’s area was shared between four intensity distributions. A dual-focusing lens with a slit was used to improve the temporal resolution of FINCH but the approach could not be extended for imaging complicated objects and had a smaller field of view [12]. One direct method of avoiding the twin image and bias terms in holography is to switch to an off-axis optical configuration in which the twin image and bias terms are spatially separated during reconstruction. This method was attempted in Hong and Kim [13]; however, the penalty paid in this case was the loss of lateral resolution as a perfect beam overlap cannot be obtained in off-axis configuration. In Liang et al. [14], the concepts of Nobukawa et al. [11] and Quan et al. [12] were applied simultaneously to achieve a single camera shot with a reasonable success. In Siegel et al. [15], a birefringent crystal lens interferometer was used to improve the temporal resolution of FINCH that was demonstrated using fluorescence microscopy. However, in all the above studies, the experimental requirements were cumbersome and a high price was paid to achieve the single-shot capability in FINCH.

Another incoherent imaging technique called coded aperture correlation holography (COACH) was developed in 2016 [16]. In FINCH, the self-interference is obtained between object waves modulated by two quadratic phase functions with different focal distances. In COACH, the self-interference is formed between object waves modulated by a quasi-random phase function and a constant phase. As a result, the imaging characteristics of FINCH and COACH are different from one another [17]. Since COACH does not have an image plane as FINCH does, the conventional Fresnel backpropagation method cannot be used for COACH. Therefore, a different approach was needed in the case of COACH. Consequently, in COACH, a one-time preliminary training became necessary, where the point spread functions are recorded at different axial planes and stored in a library. Then, the object hologram was cross-correlated with the point spread function library to reconstruct the object information at different planes. Initially, the cross-correlation was achieved using a matched filter and a phase-only filter [18], as well as three camera shots. In the later studies, it was observed that self-interference occurred within the object wave modulated by a quasi-random phase mask and therefore two-beam self-interference is redundant for recording 3D information [19]. The system without the two-beam interference was named interferenceless COACH or I-COACH [19]. In the subsequent studies, the number of camera shots were reduced to two [20] and finally to one [21] via the development of the non-linear filter.

All the above incoherent imaging systems, such as FINCH, COACH, and I-COACH, are linear imaging systems regarding intensity. Using the linearity principle, for any complicated object *O*, the intensity distribution can be given as *I_O_* = *O*⊗*I_PSF_,* where “⊗” is a 2D convolutional operator. The image of the object *O’* is reconstructed using a cross-correlation between *I_PSF_* and *I_O_*, *O*’ = I_O_*I_PSF_, where “*” is the 2D correlation operator. In the earlier studies [1,2,3,4,5,6,7], the image reconstruction in FINCH was achieved using Fresnel backpropagation and requires at least three camera recordings to synthesize a complex hologram for which the reconstructing quadratic phase function (*I_PSF_*) was optimal. However, the quadratic phase function is not an optimal function for reconstructing object information from an amplitude-only single camera recording. In the proposed method, a single camera shot becomes sufficient by adapting the non-linear filter approach of I-COACH. In summary, the similarity in the above three methods of COACH, I-COACH, and FINCH is that they are all linear systems regarding intensity such that the same reconstruction mechanism via cross-correlation can be applied to all of them. The differences in the above methods exist in the beam properties, which create the self-interference distribution and the resulting different imaging characteristics [17]. 

In this study, we proposed a new hybrid diffractive optical element (HDOE) designed by random multiplexing an axicon and a Fresnel zone lens. The HDOE is used as the only optical element for demonstrating FINCH. The proposed method is different from conventional FINCH [1] since the self-interference occurs between a conical wave and a spherical wave instead of two spherical waves. Furthermore, the proposed method uses the non-linear correlation of FINCH and so only requires a single camera shot for spatial and spectral imaging. The HDOE is sensitive to changes in both distances and wavelengths. In other words, the HDOE generates unique self-interference distributions for a particular wavelength and depth. Therefore, by recording the point spread functions for every depth and wavelength in advance, any multicolor object hologram recorded using a monochrome sensor can be decomposed into depth-specific and wavelength-specific information. This is the rationale behind the proposed idea. 

In the previous studies on spatial and spectral imaging with COACH [22], a total of 40 camera shots were required to reconstruct the spatially and spectrally resolved images. Furthermore, the technique used two spatial light modulators. The development shown in Hara et al. [23] used a wavelength multiplexing method and required two polarizers, a polarization-sensitive spatial light modulator, and involved 2N+1 recordings, where N is the number of wavelengths. In Sahoo et al. [24], a diffuser was employed and point spread function training and reconstruction were presented, just like in the current study. In References [25,26,27], the experimental setup was bulky with numerous optical components with cumbersome alignment requirements. Compared to the above methods, the proposed technique has numerous advantages as it uses only a single diffractive element without polarizers and spatial light modulators, and requires only a single camera shot. 

The modified FINCH involves two steps. In the first step, the point spread function library is recorded using a point object at different wavelengths and axial locations along the optical axis. In the next step, an object is placed within the axial boundaries of the point spread function library, illuminated using wavelengths within the spectral boundaries, and the object hologram is recorded. The non-linear correlation between the point spread function library and the object hologram reconstructs the four-dimensional image of the object in 3D space and spectrum. 

## 2. Methodology 

**Figure 1:**
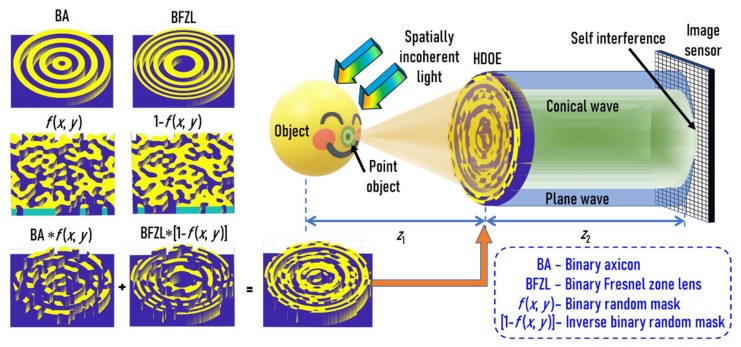
Optical configuration of Fresnel incoherent correlation holography (FINCH) with a randomly multiplexed axicon and a Fresnel zone lens. A conical wave (green) and a plane wave (blue) is generated for every object point. The two beams have the same diameter. The creation of the hybrid diffractive optical element (HDOE) from a binary axicon, a binary Fresnel zone lens, binary random matrix, and an inverse binary random matrix is shown.

The optical configuration of FINCH with the HDOE is shown in Figure 1. The object wave is incident on an HDOE, and for every spatially incoherent object point, a self-interference pattern between a mutually coherent conical wave and plane wave is generated using the HDOE, which are added up in the sensor plane. The mathematical analysis for the above system is as follows. The complex amplitude of light diffracted from a point object located on the axis at a distance of *z* from the HDOE is given as $Q\left(1/z\right)=\exp\left(j\pi r^{2}/\lambda z\right)$, where *r* is the radial coordinate and *λ* is the wavelength. The complex amplitude of the HDOE is given as $\left\{\exp\left(-j\pi r^{2}/\lambda z_{1}\right)f\left(x,y\right)+\exp\left(-j2\pi r\alpha /\lambda \right)\left[1-f\left(x,y\right)\right]\right\}$, where *α* is the angle of the axicon and *f*(*x*,*y*) is a binary random function with a scattering ratio *β*. The complex amplitude after the HDOE is given as $\left\{\exp\left[j\pi r^{2}/\lambda \left\{\left(1/z\right)-\left(1/z_{1}\right)\right\}\right]f\left(x,y\right)+\exp\left(-j2\pi r\alpha /\lambda \right)Q\left(1/z\right)\left[1-f\left(x,y\right)\right]\right\}$. The complex amplitude at the image sensor located at a distance of *z*_2_ from the HDOE is given as a convolution of the complex amplitude after HDOE with $\;Q\left(1/z_{2}\right)=\exp\left(j\pi r^{2}/\lambda z_{2}\right)$. The intensity at the image sensor is given as:

(1) $$I_{PSF}={\left|\left\{\exp\left[j\pi r^{2}/\lambda \left\{\left(1/z\right)-\left(1/z_{1}\right)\right\}\right]f\left(x,y\right)+\exp\left(-j2\pi r\alpha /\lambda \right)Q\left(1/z\right)\left[1-f\left(x,y\right)\right]\right\}⊗Q\left(1/z_{2}\right)\right|}^{2}.$$

From Equation (1), two main components that manipulate the intensity distribution are seen. The first component $f\left(x,y\right)$ is the binary random phase function attached to the residual complex amplitude $\exp\left[j\pi r^{2}/\lambda \left\{\left(1/z\right)-\left(1/z_{1}\right)\right\}\right]$ when the negative quadratic phase of the lens acts upon the quadratic phase of the incoming light. When propagated to *z*_2_, this component will generate a random diffraction pattern characteristic of the random multiplexer. The second component consists of the axicon phase and the quadratic phase with the random multiplexing function $\left[1-f\left(x,y\right)\right]$, which will result in a Bessel-like function with random multiplexing noise when propagated to *z*_2_. The intensity distribution of a complicated object can be given as *I_O_* = *O*⊗*I_PSF_*. The image of the object *O’* is reconstructed using a cross-correlation between *I_PSF_* and *I_O_*, *O*’ = *O*⊗I_PSF_*I_PSF_. Alternatively, the image can be considered as the object sampled by the autocorrelation function I_PSF_*I_PSF_. The regular cross-correlation *I*_O_*I_PSF_ is also called the matched filter [16,18]. The other versions of the matched filter, including the Weiner filter and the phase-only filter, have better performances and resolutions. A recent study concluded that the non-linear filter has the best performance in terms of the signal-to-noise ratio and resolution [28]. The reconstruction using a non-linear filter can be expressed as:

(2) $$I_{R}=\left|F^{-1}\left\{{\left|{\tilde{I}}_{PSF}\right|}^{p}exp\left[i\;arg\left({\tilde{I}}_{PSF}\right)\right]{\left|{\tilde{I}}_{O}\right|}^{q}exp\left[-i\;arg\left({\tilde{I}}_{O}\right)\right]\right\}\right|,$$

where the values of *p* and *q* are tuned between −1 to +1 until a case with minimum entropy is obtained. The entropy is expressed as $S\left(p,q\right)=-\sum \sum \phi \left(m,n\right)log\left[\phi \left(m,n\right)\right],$ where $\phi \left(m,n\right)=\left|C\left(m,n\right)\right|/\underset{M}{\sum }\underset{N}{\sum }\left|C\left(m,n\right)\right|$, (*m,n*) are the indexes of the correlation matrix, and *C(m,n)* is the correlation distribution. 

The HDOE was designed with only two levels. The self-interference is formed between two waves, namely a spherical wave and a conical wave, which are generated by a Fresnel zone lens and an axicon respectively. The binary Fresnel zone lens is multiplied by a binary random matrix with a particular scattering ratio and the binary axicon is multiplied by the inverse of the same binary random matrix. The resulting matrices are added to obtain the binary HDOE, as shown in Figure 1. The light from the point object is incident on the HDOE, which consists of two components, namely a Fresnel zone lens and an axicon. The Fresnel zone lens collimates the incident light when the object plane matches with the front focal plane of the Fresnel zone lens, while the axicon generates a Bessel-like beam. Due to the random multiplexing and the binary profile of the two elements, the generated beams are slightly different from that of the expected ones with some multiplexing noise and effects of higher diffraction orders. These two beams interfere in the sensor plane to produce a Bessel-like self-interference pattern with multiplexing noises. This is the point spread intensity distribution. For a complicated object, every independent point of the object generates their own intensity pattern, which are shifted and scaled depending upon their 3D location and are added in the sensor plane. Any change in the wavelength or depth generates a unique signature due to the wavelength- and depth-dependent diffractive effects of the HDOE. 

## 3. Experiments and Results

**Figure 2:**
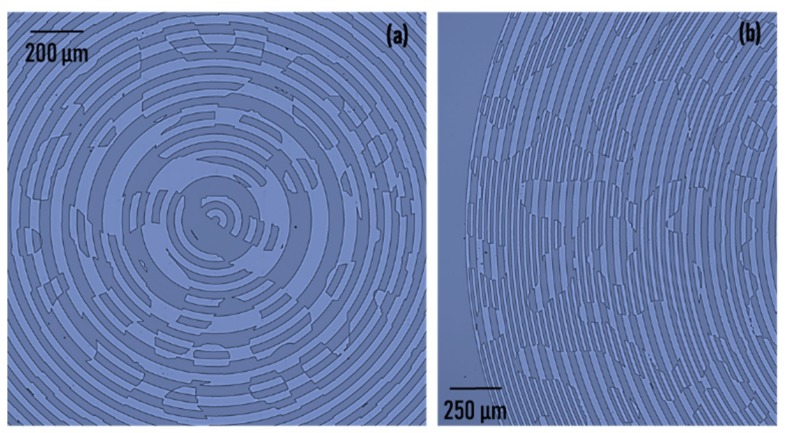
Optical microscope images of the (**a**) central and (**b**) outermost part of the HDOE fabricated using electron beam lithography. The dark blue color indicates the resist remained and the light blue color indicates the resist was removed.

The HDOE was designed for a wavelength of *λ* = 617 nm and a diameter of 5 mm. The focal length of the Fresnel zone lens was *f* = *z*_1_ = 10 cm. The angle of the axicon was approximately *α* = 0.6° with a period of Λ ≈ 60 μm. The random phase function *f*(*x*,*y*) was synthesized using the Gerchberg–Saxton algorithm with *β* = 0.1 radians [8,20]. The HDOE was designed as a binary element with only two-phase levels. The HDOE was fabricated using electron beam lithography (EBL; Raith 150^2^, RAITH, Dortmund, Germany) in a PMMA 950K (A7) resist (Microchem, Round Rock, TX, USA) on indium tin oxide (ITO)-coated glass substrates with a thickness of 1.1 mm and developed using methyl isobutyl ketone (MIBK) and isopropyl alcohol (IPA) solution (Microchem, Round Rock, TX, USA). The electron beam dose was 150 μC/cm^2^. A write field of 100 μm was used with stitching to fabricate a 5 mm size element. The optical microscope images of the HDOE are shown in Figure 2a,b. The results indicate no stitching errors. The thickness of the PMMA resist was found to be around 700 nm, which was close to the expected value of *λ*/2(*n_r_* − 1) = 617 nm (*n_r_* is the refractive index of the resist ≈ 1.5), and achieved the maximum efficiency of 40.5% for a binary element. The outermost zone width was approximately 12 μm. However, the random multiplexing generated features smaller than 12 μm at various locations. 

**Figure 3:**
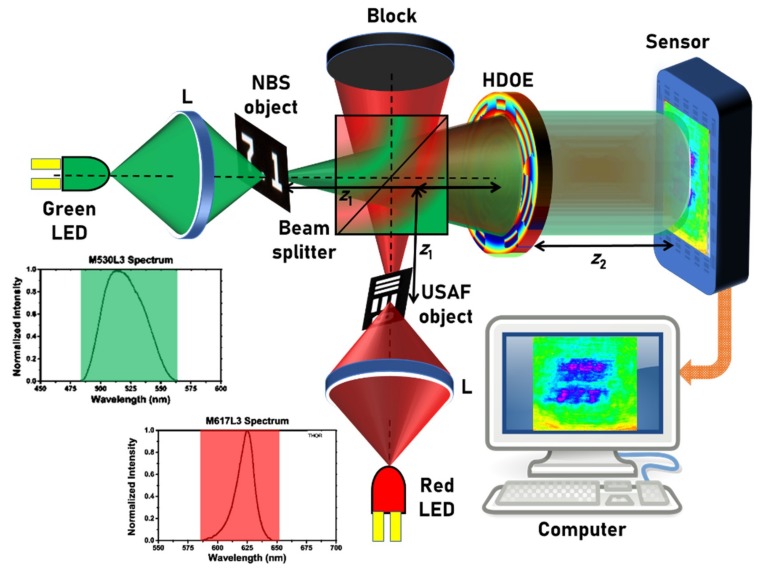
Schematic of the two-channel experimental setup. NBS: National Bureau of Standards, USAF: United States Air Force.

The experimental demonstration was carried out using two LEDs (M617L3, *λ_c_* = 617 nm, full width at half maximum (FWHM) = 18 nm; M530L3, *λ_c_* = 530 nm, FWHM = 33 nm), which were spatially incoherent and had low temporal coherence owing to the larger values of the FWHMs. It must be noted that the previous studies [1,2,3,4,5,6] were carried out with temporally coherent sources with a FWHM of 1 nm. A pinhole with a diameter of 20 µm was used to simulate the point object. Two standard objects, namely the United States Air Force (USAF) resolution target (group 2, element 6, 7.13 lp/mm) and the National Bureau of Standards (NBS) resolution target (7.1 lp/mm), were used to generate two plane objects with different thicknesses and wavelengths. The schematic of the two-channel experimental setup is shown in Figure 3. The light from the two LEDs were collected using two identical lenses L with a focal length of 10 cm that critically illuminated the two objects. The light that formed the two channels were combined using a beam splitter (BS). The light that diffracted from objects was modulated using the HDOE and the self-interference intensity distribution was recorded using an image sensor (Thorlabs DCU223M (Thorlabs, Newton, NJ, USA), 1024 × 768 pixels, pixel size = 4.65 μm). 

**Figure 4:**
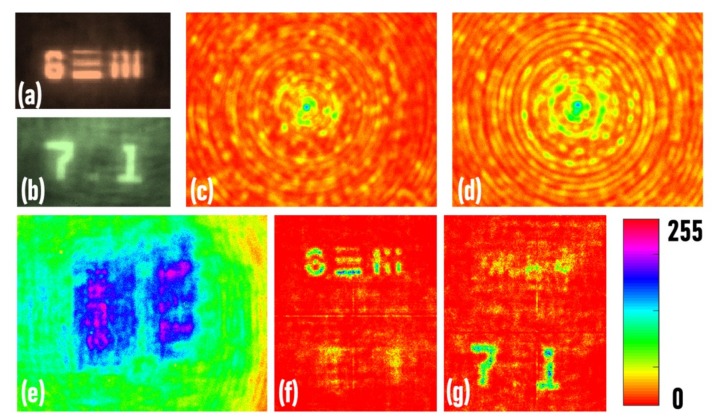
Direct imaging of the (**a**) USAF and (**b**) NBS objects. Point spread holograms recorded for (**c**) *λ_c_* = 617 nm and (**d**) *λ_c_* = 530 nm. (**e**) Object hologram recorded when the USAF and NBS objects were at the same distance from the HDOE. (**f**,**g**) The reconstruction results using (**c**) and (**d**), respectively. The scale corresponds to the pixel intensity recorded by the image sensor.

In the first experiment, the direct images of the two objects were recorded, as shown in Figure 4a,b, respectively. The images of the red and green point spread holograms were recorded at a distance of *z*_1_ = 10 cm and *z*_2_ = 10 cm, as shown in Figure 4c,d, respectively. In the next experiment, the two objects (USAF and NBS objects) were mounted at the same distance *z*_1_ = 10 cm and critically illuminated using red and green LEDs, respectively. The image of the hologram is shown in Figure 4e. The reconstruction results (*p* = 0, *q* = 0.63) of the hologram using the red and green point spread functions are shown in Figure 4f,g, respectively. It is seen that the point spread holograms only successfully reconstructed the objects illuminated by the same wavelength, while the object illuminated by a different wavelength was not reconstructed. 

**Figure 5:**
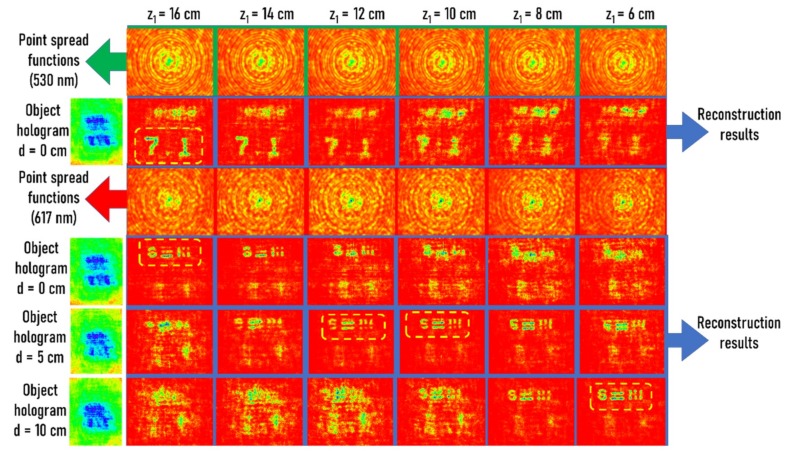
Four-dimensional reconstruction results demonstrated using holograms of two plane objects of different thicknesses (*d* = 0, 5, and 10 cm) and two different wavelengths. The point spread functions reconstructed the depth-specific and wavelength-specific information with the maximum focus while the information from other planes and wavelengths were blurred with decreased intensities. The blue border lines indicate the reconstruction results. The green border lines and red border lines indicate the point spread function of the green and red wavelengths and the yellow square boxes indicate the cases with the best focus.

In the final experiment, the four-dimensional imaging capabilities were evaluated by changing the relative distances between the two objects. In this way, the recorded holograms contained spatially and spectrally varying information across it. The distance between the NBS and USAF objects along the *z*-direction was varied using *d* = 0, 5, and 10 cm and the corresponding holograms were recorded. The holograms were reconstructed using point spread functions of the two wavelengths 530 nm and 617 nm recorded at *z*_1_ = 6 cm to 16 cm in steps of 2 cm. From the reconstruction results shown in Figure 5, it was observed that the system could distinctly resolve the axial and spectral information. 

## 4. Discussion

Axicon has a high focal depth when compared to a lens; therefore, the replacement of the quadratic phase with a conical phase in FINCH is expected to increase the focal depth or decrease the axial resolution of the imaging [29]. In the proposed configuration, there were two factors, namely *f*(*x*,*y*) and $\;\exp\left(-j2\pi r\alpha /\lambda \right)$ in Equation (1), that could be tuned to compensate for or enhance one another to produce desired behavior in the lateral and axial resolutions. In the current configuration, the effect of the random multiplexing function *f*(*x*,*y*) overshadowed the effect of the conical phase, increasing the axial resolution. This is a surprising result since from the hologram in Figure 4e, the object information (7.1) was clear just by looking into the hologram, which is a characteristic of the conical phase but the random function *f*(*x*,*y*) dominated, resulting in a high spectral resolution. The same observation is seen in Figure 5. The point spread functions recorded for the two wavelengths at different distances did not seem to vary much owing to the effect of the conical phase. However, the fine variations of the randomness dominated the axial and spectral resolutions of the system. However, further studies are needed to fully understand the proposed imaging technique. 

## 5. Summary, Conclusions, and Outlook

FINCH was demonstrated using a modified approach using a diffractive axicon instead of a diffractive lens. A random multiplexing method was used to combine the two phase functions in a single diffractive element. A modified procedure for recording and reconstructing holograms was proposed and demonstrated. The resulting imaging technique could resolve objects in 3D space using a spectrum from a single camera shot. Therefore, including time, the imaging technique could resolve events in five dimensions. This is a notable advancement in the area of incoherent multicolor imaging when compared to the existing and state of the art spatial and spectral imaging techniques [22,23,24,25,26,27]. In Vijayakumar and Rosen [22], the signal-to-noise ratio was much lower than the developed method and therefore about 40 camera shots were needed for statistical averaging. The technique in Hara et al. [23] requires more camera shots and active devices, such as SLM, but does not require recording the PSF library and offers a better signal-to-noise ratio. The technique described in Sahoo et al. [24] requires only a single element as the proposed method but suffers from a limited field of view and 3D spatial imaging was not demonstrated. The techniques in References [25,26,27] do not require recording the PSF library but require many optical elements and camera shots.

A recently accepted manuscript on spatial and spectral imaging with incoherent light and coherent computational superposition [30] was brought to our attention. The technique does not require one-time PSF training, in contrast with the proposed method, and the reconstructed images have an excellent signal-to-noise ratio that is on par with that of direct imaging. However, the technique requires an active device, such as a spatial light modulator and many optical components, and requires multiple camera recordings.

In this preliminary study, a new possibility of spatial and spectral imaging was demonstrated using FINCH. We believe that what is seen in this study is an introduction to this method and further studies are needed to understand the hidden capabilities of the method. Some of the areas in the study that are interesting for further research are as follows. First, in FINCH, the super-resolution is only exhibited at a specific plane. Therefore, to image different planes of a thick object, a manual stage movement is necessary to match that particular object plane to the super-resolution hologram plane. In the proposed configuration, the super-resolution capability might be simultaneously available for a range of depths owing to the imaging characteristics of the axicon. Second, it will be interesting to study the imaging characteristics by trading off the scattering degree of the multiplexer with the angle of the axicon. A new direction has been set to explore the capabilities of FINCH by replacing the lens with an axicon. It will be interesting to study the performances by employing exotic beams, such as airy beams, beams carrying an orbital angular momentum, and structured light. These interesting research directions are beyond the scope and space limitations of this short communication.
